# Photocurable Polymer-Based 3D Printing: Advanced Flexible Strain Sensors for Human Kinematics Monitoring

**DOI:** 10.3390/polym15204170

**Published:** 2023-10-20

**Authors:** Christopher Billings, Ridwan Siddique, Yingtao Liu

**Affiliations:** 1 School of Aerospace and Mechanical Engineering, University of Oklahoma, 865 Asp Ave., Norman, OK 73019, USA; 2 Norman North High School, 1809 Stubbeman Ave., Norman, OK 73069, USA; rsiddique770@gmail.com

**Keywords:** additive manufacturing, 3D printing, photocurable polymer, flexible sensor, strain sensor, piezoresistivity, wearable device

## Abstract

Vat photopolymerization-based additive manufacturing (AM) is critical in improving solutions for wearable sensors. The ability to add nanoparticles to increase the polymer resin’s mechanical, electrical, and chemical properties creates a strong proposition for investigating custom nanocomposites for the medical field. This work uses a low-cost biocompatible polymer resin enhanced with multi-walled carbon nanotubes (MWCNTs), and a digital light processing-based AM system to develop accurate strain sensors. These sensors demonstrate the ability to carry a 244% maximum strain while lasting hundreds of cycles without degradation at lower strain ranges. In addition, the printing process allows for detailed prints to be accomplished at a sub-30 micron spatial resolution while also assisting alignment of the MWCNTs in the printing plane. Moreover, high-magnification imagery demonstrates uniform MWCNT dispersion by utilizing planetary shear mixing and identifying MWCNT pullout at fracture locations. Finally, the proposed nanocomposite is used to print customized and wearable strain sensors for finger motion monitoring and can detect different amounts of flexion and extension. The 3D printed nanocomposite sensors demonstrate characteristics that make it a strong candidate for the applications of human kinematics monitoring and sensing.

## 1. Introduction

Recent advances in additive manufacturing (AM) have enabled the development of personalized medical devices and wearable sensors for practical, inexpensive, and reliable point-of-care monitoring and diagnostics [1,2,3]. Advanced AM technologies, often also referred to as 3D printing, have been integrated with materials science for the creation of novel solutions to design and fabricate customized, intricate structures and devices that can conform comfortably to the human body, offering enhanced wearability and improved monitoring accuracy. Traditional manufacturing methods, such as casting and injection modeling, are often restricted by their adaptability, scalability, and high costs. However, advanced 3D printing technologies offer unique advantages in tailoring sensor designs for individual needs. Recent studies have showcased wearable sensors that can track an array of biomechanical movements, from the subtle flexion of a finger to the dynamic motions of limbs [4,5,6]. These sensors, manufactured using 3D printing techniques, are rapidly transforming the domains of sports analytics, healthcare monitoring, and rehabilitative therapy, emphasizing the technological synergy between 3D printing and wearable devices.

The most widely recognized polymer AM technologies are material extrusion-based methods, such as fused deposition modeling (FDM) and direct ink writing (DIW) [7,8,9]. Particularly, FDM-based polymer AM is well-recognized for the 3D printing of thermoplastic polymers, such as polylactic acid (PLA), acrylonitrile butadiene styrene (ABS), polyurethane (TPU), and polyvinyl alcohol (PVA) because of its cost-effectiveness, ease of use, and widespread accessibility [10,11,12]. However, 3D printing of these thermoplastic polymers using the FDM method often faces challenges, such as poor layer adhesion, spatial limited resolution, low surface smoothness, and potential warping of printed parts, especially with polymers with high thermal expansion [13]. Additionally, most of these materials have relatively high elastic moduli at room temperature and low biocompatibility. Therefore, 3D printing of these materials using FDM-based AM is inappropriate for developing flexible and wearable sensors. DIW-based polymer AM has been employed to 3D print wearable sensors using highly flexible and biocompatible silicone rubbers, such as polydimethylsiloxane (PDMS) and Ecoflex silicones [14,15,16,17]. Using DIW-based AM technologies, PDMS can be integrated with other conductive nanoparticles to improve the sensing range and sensitivity of the 3D printing wearable sensors and even enable the detection of electrical and thermal signals [18]. However, ensuring consistent rheological properties in PDMS inks is crucial to maintaining the integrity of printed structures [19]. Variations can lead to printing defects or discrepancies in sensor performance. Also, post-processing or curing methods are necessary to finalize the structure and properties of the printed PDMS, which can sometimes alter the desired properties or the dimensional accuracy of the printed device. Vat photopolymerization-based AM, such as stereolithography (SLA) and digital light processing (DLP) techniques provide notable advantages in developing flexible and wearable sensors due to their high surface smoothness, excellent precision, and optimal mechanical and electrical properties [20,21,22]. SLA/DLP-based printing, which uses light to cure liquid resins layer by layer, offers exceptional spatial resolution and accuracy, allowing for fabricating intricate sensor designs with superior mechanical properties. Additionally, the diversity of photopolymer resins available for SLA/DLP processes ensures optimal flexibility, making them particularly suited for flexible and wearable sensors that require close conformance to body contours without compromising functionality. For example, Su et al. employed SLA-based 3D printing for the rapid prototyping of flexible sensors with optimal geometries and high sensitivity, which could be potentially used for the monitoring of human body fluids, and various Internet of Things applications [23]. Due to the limited electrical properties of polymers, nanoparticles are often required to tailor key electrical properties of polymers, leading to the development of novel nanocomposites with optimal sensing performance.

The integration of nanoparticles within 3D printed polymers has been demonstrated to be an effective approach to improving the performance of wearable sensors. A broad range of nanoparticles, such as single-walled carbon nanotubes, multi-walled carbon nanotubes (MWCNTs), carbon nanofiber, graphene, silver nanowires, and gold nanowires, have been investigated as a transformative approach in the realm of wearable and flexible sensor development [24,25]. The uniform dispersion of a small amount of such nanoparticles (0.5–2 wt.%) can significantly increase the electrical conductivity of obtained polymer nanocomposites by creating a conductive network within polymers, resulting in the piezoresistive-based sensing capability for flexible and wearable sensors. Among the vast array of nanoparticles, MWCNTs have been recognized as one of the most promising nanoparticles for wearable sensor development due to their superior electrical conductivity, high aspect ratio, and strong mechanical strength [26]. When uniformly dispersed and embedded within 3D printed polymer nanocomposites, MWCNTs foster the creation of highly sensitive, durable, and responsive sensors that are capable of detecting physiological signals or environmental changes due to the tunneling effect in the MWCNT network [27]. The synergy between 3D printing, polymers, and MWCNTs have the potential to drive remarkable advancements in the wearable technology sector, underscoring the transformative potential of nanoscale enhancements. 

Recently, researchers have been investigating the integration of nanoparticles within UV curable resins for the 3D printing of wearable sensors using SLA/DLP-based AM methods [28]. For example, Yin et al. employed DLP-based 3D printing of ionically conductive hydrogels for the development of capacitive sensors which were capable of detecting both static and dynamic pressures and strains [29]. Guo et al. developed highly flexible nanocomposites using SLA-based 3D printing technology and a reduced functionalized graphene oxide/acrylate nanocomposite [30]. Although photocurable polymer materials allow for intricate sensor design, they also introduce several challenges, including the incomplete cure after 3D printing, poor mechanical properties, unstable manufacturing conditions and qualities, and complicated post-processing. Various fabrication and process parameters, including post-curing temperature and time, can significantly influence a sensor’s ultimate performance [31,32]. The curing level is primarily affected by the applied temperature profile, including the heating and cooling rates and heating time. Therefore, elevated chamber temperatures have proven to improve the mechanical properties of the fabricated sensors owing to enhanced curing [33]. These manufacturing parameters create a complex manufacturing environment that should be carefully investigated for repeatable results.

In this paper, a DLP-based 3D printing and post-processing approach was investigated for the development of highly flexible, reliable, and wearable sensors using MWCNTs and photocurable resins. The 3D printing and post-processing parameters were optimized to improve the printing quality and repeatability. The mechanical, electrical and sensing functions were studied systematically. The performance of the developed wearable sensors was characterized by monitoring the finger motions, mimicking the grasp motion of human hands. 

## 2. Materials and Methods

### 2.1. Materials

Unless otherwise stated, all materials and reagents were used as received. The MWCNTs were purchased from Sigma Aldrich with diameters of 50–90 nanometers and an aspect ratio of 100. The photocurable thermoset Superflex resin was purchased from 3D Materials. 

### 2.2. Materials Preparation and 3D Printing

The photocurable thermoset matrix and MWCNTs were combined by a concentration of 0.25 wt.% fillers to the matrix for the preparation of the novel nanocomposites that could be processed by DLP-based AM process. The MWCNTs were uniformly dispersed in the polymer resin by mixing the nanoparticles using a planetary Thinky AR100 mixer (Laguna Hills, CA, USA) for a time span of 10 min in batches of 70 mL. After the mixing process, the material’s ending temperature was 37 °C.

An SLA 3D printer (Elegoo, model: Mars 2 Pro, Shenzhen, China) was employed to 3D print all samples and wearable sensors with custom curing settings. All samples were printed following ASTM D638 Type V samples with a width of 9.525 mm, gauge length of 63.5mm, and thickness of 7.625 mm [34]. The original equipment manufacturer (OEM) of the SLA 3D printer recommended the cure time for the untreated nanocomposite utilizing the 50-micron layer height with 20 s for the base layers and 9 s for all the subsequent layers. With a 50-micron layer height, the tested print settings were 40 s for the base layer and 7.5 s for all the subsequent layers. One key improvement in this study was that the printer was placed inside a custom convection heated chamber at 40 °C to help control resin viscosity and degree of cure. The elevated 3D printing temperature facilitated a faster curing process, which was demonstrated during the normal curing setting. The increased bottom layer curing time was employed to increase the degree of cure of the bottom layer because the increased chamber temperature also reduced layer stiffness during printing. 

Once the samples were successfully printed, they were removed from the heated chamber and cooled completely before removing from the build plate. There was no geometry change due to post stress relief and still fell within ASTM D638 size tolerances. The samples were cleaned using isopropyl alcohol, the recommended cleaning agent for this polymer matrix. Once cleaned, they were immersed in a water-filled glass container and then post-cured on a rotating platform inside a curing oven, using a 405 nm light at 60 °C for an hour. The post-curing of MSLA 3D printed samples could reduce any potential warping of the samples due to any residual stress generated during the AM process. 

### 2.3. Mechanical, Thermal, and Rheological Testing of 3D Printed Samples

In this study, multiple mechanical, thermal, and rheological tests were conducted to fully understand and showcase the applications of the newly developed nanocomposite and its 3D printing technique. First, we investigated the material’s ultimate tensile strength, Young’s modules, and maximum elongation to identify the material’s maximum working conditions. Second, cyclic tests were carried out to demonstrate the long-term performance and related electrical resistance response of the 3D printed sensors. Third, we conducted viscosity testing to understand the effect of the elevated printing temperature on the material. Fourth, samples were analyzed under a differential scanning calorimeter to determine the glass transition temperature of the developed nanocomposite materials. Optical microscopic imaging was employed to evaluate MWCNT dispersion and alignment. Lastly, a practical application is demonstrated for use in human-based sensors.

Nanocomposite dogbone samples were printed following ASTM D638 Type V dimensions, ensuring the largest number of samples could be printed on the entire building platform. These printed samples were used for tensile and fatigue tests using an Instron 5969 with a 5 kN load cell. A Hioki RM3545 resistance meter was employed for the measurement of electrical resistance and the recording of piezoresistive sensing data in this study. Tensile tests were performed using the load rate of 10 mm/min, while all the fatigue tests were conducted at the strain rate of 20% strain per minute.

To minimize contact electrical resistance, copper strips were glued on the 3D printed strain sensors using an in-house developed conductive nanocomposite paste consisting of PDMS and MWCNTs at a 2% weight concentration. The applied paste ensured that the electrical resistance would be significantly smaller than the printed samples as it had 8 times the amount of MWCNT content. Therefore, all the measured electrical resistance variations were due to the applied strain to the 3D printed nanocomposite sensors. Additionally, the location in which the conductive paste was applied was isolated from resistive measurements during testing.

The viscosity of the prepared nanocomposites was tested by an NDJ-9S rotary viscometer. Nanocomposite was tested by measuring the torque required to rotate a spindle within the sample. The resulting data provided critical insights into the nanocomposite’s rheological behavior. By subjecting nanocomposites to varying shear rates and temperatures, a comprehensive understanding of how these materials respond to different conditions were identified. As the heating process was performed on a hot plate instead of inside the oven for this test, it took several minutes for the nanocomposites to reach thermal equilibrium.

## 3. Results and Discussions

### 3.1. Mechanical Performance of 3D Printed Nanocomposite Samples 

**Figure 1:**
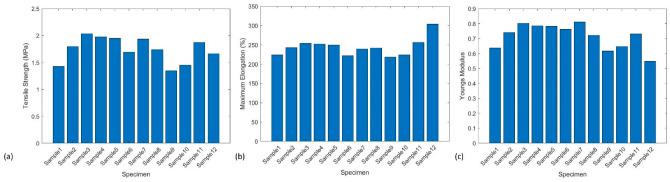
(**a**) Ultimate tensile test results of ASTM D638 Type V samples; (**b**) maximum elongation of the tested nanocomposites before failure; (**c**) Young’s modulus of the tested nanocomposites.

Critical mechanical properties, including tensile strength, max elongation, and Young’s modulus under tensile loads, were tested using 12 nanocomposite samples. The experimental results showed that the average strength, maximum elongation, and Young’s modulus were 1.74 ± 0.084 MPa, 244% ± 23.2%, and 0.715 ± 0.084 MPa, respectively. Figure 1a shows the samples’ ultimate tensile strength results. Figure 1b demonstrates the maximum elongation capabilities of this material as it could be stretched up to 244% of its original length on average. The Young’s module of the developed nanocomposite is shown in Figure 1c. Since the employed polymer was a type of flexible thermoset resin, it is expected to obtain the low Young’s modulus in the 3D printed nanocomposite samples. 

According to the material data of the virgin polymer provided by the supplier, maximum elongation of virgin polymer was 80% strain [35]. The fully cured and 3D printed nanocomposite obtained the maximum elongation of 244%. The increased maximum elongation of the nanocomposite could be due to the MWCNT alignment along the gauge direction of the tested sample. 

### 3.2. Cyclic Tensile Tests for Sensor Characterization

**Figure 2:**
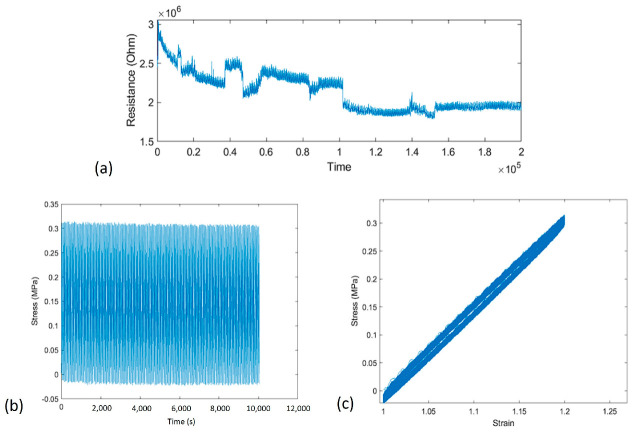
(**a**) Variation of electrical resistance of 3D printed nanocomposite over time under cyclic tensile load; (**b**) tensile stress of the sample over time; (**c**) stress-strain history of the sample for all the 250 cycles.

To properly characterize the sensing capability of the 3D printed nanocomposite, cyclic tensile tests were conducted. The sample tested was set up to run a continuous cyclic test of 20% maximum tensile strain for 250 cycles. The variation of electrical resistance was continuously monitored and recorded during the entire cyclic tests. The electrical resistance over time, stress over time, and stress-strain curves can be seen in Figure 2.

The maximum tensile strain of 20% was applied to all cycles. As observed in Figure 2, the stress of the sample remained consistent with no obvious degradation of strength. It is noted that the electrical resistance response of the sample stabilized after the first 125 cycles, then remained stable for the remainder of the tests. However, it was observed that the total electrical resistance decreased in the first 125 cycles. This was possibly due to the sample warming up as it cycled and reached equilibrium. 

### 3.3. Characterization of Piezoresistance-Based Sensor Performance 

**Figure 3:**
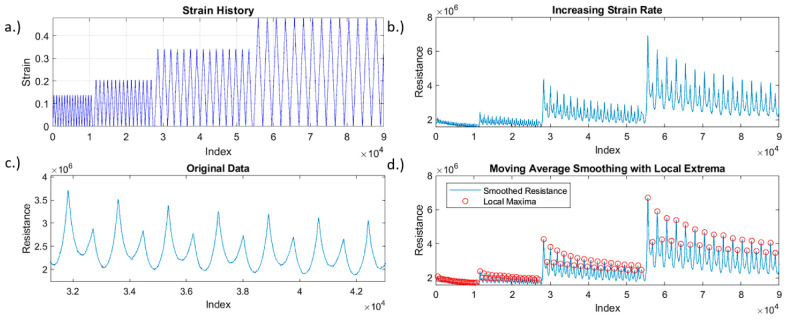
(**a**) Applied cyclic loads; (**b**) piezoresistive resistance responses at multiple different maximum strains; (**c**) typical sensor signal up to 35% tensile strain; (**d**) smoothed data with local extrema automatically detected.

A key feature of a highly adaptable strain sensor is its capacity to distinctly differentiate various levels of strain via unique electrical resistance responses. To characterize the performance of 3D printed strain sensors, a short cyclic test was set up with varying strain levels (Figure 3a). Then, the strain was increased in a step-like function until the sample failed and the measured sensor signals are shown in Figure 3b,c. Finally, the recorded sensor data was analyzed using in-house developed MATLAB code by employing a moving average smoothing technique and local extrema function. The analytical results demonstrated how these sensors’ output could be analyzed in real-time and movements automatically characterized, as shown in Figure 3d. 

The sensitivity of the 3D printed strain sensors was evaluated by calculating its gauge factor. Typically, gauge factor is a measure of how much the electrical resistance of a sensor changes in response to the applied mechanical strain. Mathematically, gauge factor is defined as follows:

(1) GF=∆RR0ε

where ∆R is the change in electrical resistance due to strain, $R_{0}$ is the initial electrical resistance of the sensor, and ε is the applied strain. In this study, the gauge factor for the tested sample was around 1.0 at or under 30% maximum strain. However, when the maximum tensile strain increased above 30%, the gauge factor began to increase to more than 2.0, indicating the nonlinear electrical resistance response with the applied strain. It is noted that the significant increase in the electrical resistance response at lower strain levels could be easily identified by software, therefore, more detailed signal processing and analysis could result in more precise location information. 

In this study, we developed a sensor signal processing code using MATLAB R2023b for data analysis including a moving average based on a window of 75 samples. This signal processing approach was applied to the raw electrical resistance data due to the noise experienced from the high levels of resistance. Reducing the window size could lead to more accurate deformation data but would be more difficult to sort into predetermined categories based on local extrema. Local extrema were identified by optimizing the boundary requirements to automatically capture large spikes in electrical resistance or drop offs due to the connection of the sensor. As shown in Figure 3d, the built-in maxima and minima functions were employed to establish a matrix of local extrema that could be overlayed on top of the smoothed data. 

### 3.4. Viscosity

**Figure 4:**
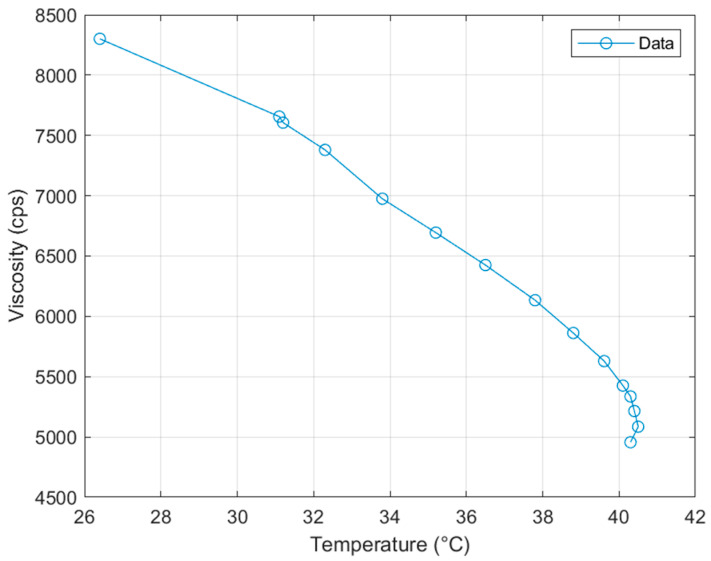
Viscosity of the polymer resin while heated to printing temperature.

It has been reported that the increased process temperature and temperature modulation of polymer resins can lead to the improved 3D printing quality and speed for DLP-based AM process [33]. However, the DLP process parameters should be optimized because of the resin’s decrease in viscosity and energy needed to cure during the exothermic reaction. Additionally, MWCNTs have been shown to absorb UV light, it is urgently needed to understand the degree of cure of MWCNT reinforced UV curable resin and optimize the print time for the base layer and the subsequent layers [36,37]. In this study, the nanocomposite and DLP-based 3D printer were both heated to 40 °C using an in-house developed chamber. As shown in Figure 4, the increased 3D printing temperature led to the 50% reduction of viscosity for the unprinted polymer resin, thereby, this approach could significantly improve the flow characteristics of the uncured nanocomposites and the printing capability.

When the uncured nanocomposite was heated, it took several minutes for the viscosity of material to equalize because adequate time was required for the material to reach thermal equilibrium. The average viscosity recorded at operating temperature was 4742 centipoises. This was still relatively high for DLP-based resin printing. Therefore, future research studies should investigate if printing can continue at higher temperatures. The development of novel polymer resin can be another solution to increase the printability. Additionally, the identification of the equilibrium between in situ print rigidity and reduction in the cure rate would be required to allow for fine detail printing without print failures. At the current temperature and viscosity of the material, the DLP-based 3D printing could be completed at a higher speed than recommended by the resin manufacturer. If higher MWCNT loadings are required, it is highly possible to require increased processing temperature for the 3D printing at the same rate. 

It is also noted that the planetary shear mixer was another important process that relied on the viscosity of the material for the development of novel nanocomposites. Uniform dispersion of MWCNT nanoparticles and the reduction of the matrix viscosity were beneficial. Therefore, it would be ideal to mix materials for a long duration to lower the viscosity to that of the printing viscosity. In this study, this goal was achieved by mixing the material for different intervals until the final nanocomposite was removed from the mixer at operating temperature due to friction-induced thermal gain. 

### 3.5. Characterization of Glass Transition Temperature

**Figure 5:**
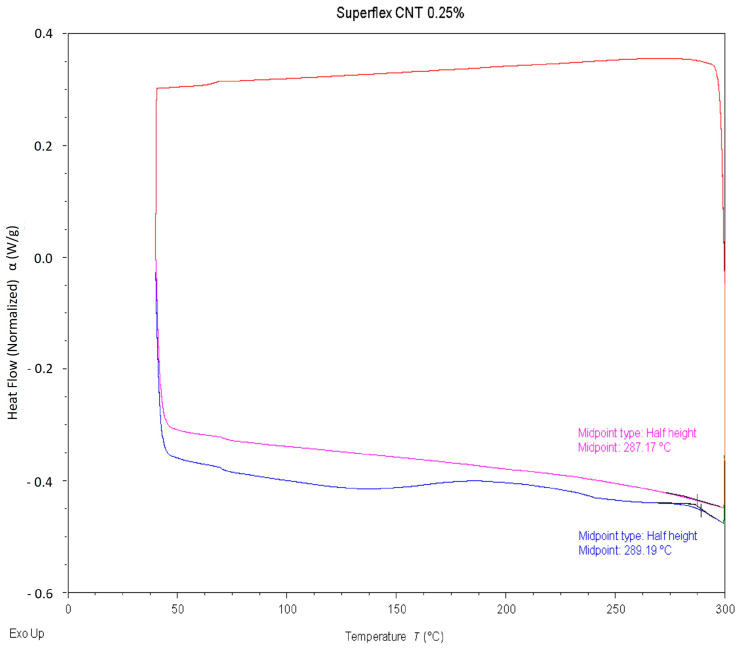
Normalized heat flow compared to temperature with glass transition temperature determined using a half-height midpoint calculation.

Characterization of the nanocomposite’s glass transition temperature would allow the material to be used in a thermal cleaning scenario for wearable sensor applications. Differential scanning calorimetry (DSC) tests were performed to determine the average glass transition temperature of the polymer resin. Since the material was determined to be amorphous, the glass transition temperature was characterized using a half-height midpoint calculation, as shown in Figure 5.

The average glass transition temperature of 288.15 °C with a standard deviation of 1.22 °C was recorded from eight different readings. Usually, traditional sterilization uses in an autoclave takes place at 121–132 °C. This result indicated that the developed 3D printable polymer and nanocomposites were well above the temperature needed for thermal cleaning systems often used for the sanitation of wearable sensors. Therefore, the developed nanocomposites were a strong candidate for prosthetic interfaces and other wearable sensing and even potential medical device applications. It is worth noting that the samples were run through two DSC cycles, and the glass transition temperature on the second cycle was often 1 °C lower than that in the first cycle. This is likely due to some sample degradation during the first cycle, changing the heat flow required for the second cycle. 

### 3.6. Microscopic Imaging and Characterization of Surface Quality

**Figure 6:**
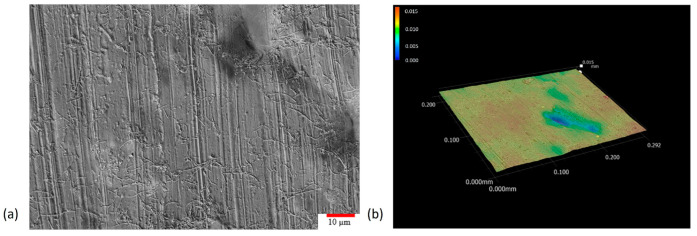
(**a**) Optical microscopic image of 2500× magnification on as printed surface with MWCNT dispersed throughout; (**b**) 3D model generated of the nanocomposite sample after fracture demonstrating the fracture failure mode.

Surface quality on 3D printed wearable sensors is crucial because it directly impacts the sensor’s performance, comfort, and durability. A smooth and defect-free surface ensures consistent and accurate sensing, minimizes skin irritation when worn for extended periods, and prevents premature wear or failure due to surface anomalies. Furthermore, a high-quality surface finish can enhance adhesion and integration with other materials or electronic components in the wearable device. In this study, a Keyence VHX-7000 ultramicroscope (Osaka, Japan) was employed for the microscopic imaging of the sample’s surface and fracture location. MWCNT dispersion, entanglement, and pullout from polymer matrix could be observed through high resolution microscopy images, too. As shown in Figure 6, the material’s surface was also mapped in 3D to provide insight into the surface finish, defects, and tear-out. 

**Figure 7:**
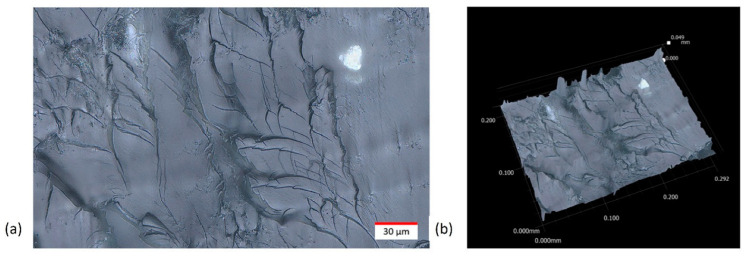
(**a**) High magnification stitched image showing the MWCNT tear out across the fracture plane; (**b**) 3D model of the fracture plane in the sample.

The obtained optical microscopic images reinforced the decision to utilize planetary shear mixing technology as the dispersion method in this study, as all the images showed a uniform dispersion of MWCNTs within UV curable and 3D printable polymer resin. MWCNT entanglement also appeared to be minimal, with many visible long strands of MWCNTs above 10 microns in length. The MWCNT supplier stated that the employed nanoparticles should have an aspect ratio above 100, and the image results demonstrated that the MWCNTs remained unbroken as their aspect ratio was often above the 100 range. Additionally, there was no MWCNT alignment in the horizontal axes, which indicated that the nanocomposites should exhibit isotropic properties in the horizontal plane. However, particle alignment was noted in the tear-out samples parallel to the horizontal axis, as shown in Figure 7. This was possibly caused by the DLP-based 3D printing mechanism pressing down on the nanocomposite of each layer and assisting the MWCNTs lay flat. This phenomenon would increase the strength of the 3D printed nanocomposites when pulled in the horizontal plane instead of the vertical axis, as shown in the 3D fracture image in Figure 7a. Very few MWCNTs were parallel to the sample surface, and instead, locations of MWCNT tearing along the main fracture lines were evident. 

When analyzing the horizontal cross-section from the vertical axis perspective, there appeared to be larger MWCNT agglomerations based on the printed layer level. While MWCNTs were prevalent on all layers, further investigation should be conducted to better understand the effects of MWCNT settlement over time, leading to varying volume ratios of employed nanoparticles for longer prints. It is noted that the top left corner of Figure 7a most prominently displayed the large MWCNT agglomerations that appeared to transmit most of the load during tension. The major fracture running down the middle of the image also had multiple locations of nanoparticle tear out evident. Further investigation into the effects of layer heights on nanoparticle dispersion and settlement could determine the impact of the printing system’s mechanical motion on aligning MWCNT in the horizontal plane. 

### 3.7. Human Kinematics Monitoring and Sensing

**Figure 8:**
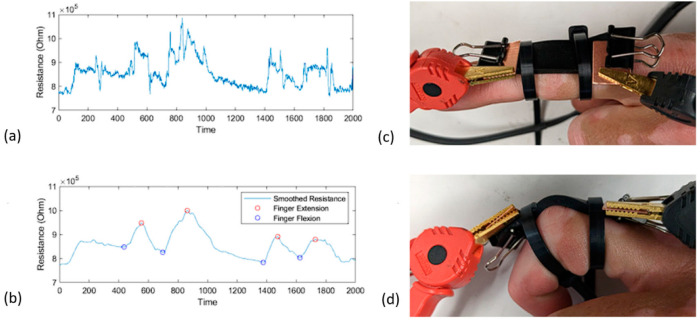
(**a**) Raw sensing data recorded by the electrical resistance meter; (**b**) MATLAB processed sensor data with automatically determined key points; (**c**) finger under extension with a mounted sensor; (**d**) finger under flexion with a mounted sensor.

While the AM processed nanocomposites have shown numerous favorable characteristics for adaptation into the wearable sensor industry, they must still perform adequately when applied to human kinematic motion monitoring and sensing systems. To analyze their capability of being skin-attached wearable strain sensors, an in-house developed prototype was attached to a human finger and employed to measure the flexion and extension motions of the proximal interphalangeal joint. The goal of this study was to determine if the hand was attempting to grip an object or was loose and relaxed. As shown in Figure 8, the signal process code developed using MATLAB could automatically smooth the raw sensor data and determine when the finger was extended or under flexion based on local extrema. This code utilized the same smoothing functions and local extrema matrix produced as described in Section 3.3. 

The finger flexion angle was easily determined by the first two peaks in Figure 8b. Since the finger was fully bent inward, the finger was only under 50% flexion in the second two peaks. As the sensor was mounted on a moving object with lots of micro-strains, the data required cleaning for accurate motion detection. For this scenario, a simple moving average was applied where the window size could be easily changed depending on the sensitivity of the resistance meter. Additionally, because the applied wearable sensors were 3D printed, each of them could be customized and designed following the hand and finger dimensions of individual user. As shown in Figure 8c, the printed sensor was designed to best fit the user’s finger joint with enough excess as the electrodes for resistance meter measurements. The only part of the sensor under flexion was the center of the skin-attached sensor between the two attachment points; therefore, the overall strain of the sample remained low. This was ideal since the overall lifespan of the wearable sensors could increase when the sample’s average strain remained low. 

Final application prints could incorporate the wire or tape electrodes inside the 3D printed wearable sensors for improved and durable connections with extended service lives. This can be achieved by modifying the printing code to pause at a certain layer and increasing the layer height to 100–200 microns, depending on the dimensions of the embedded electrodes. Another possible manufacturing method for the integration of metallic electrodes would be to utilize a low-resistance thermoplastic and press the wires and tape electrodes onto the samples with appropriate pressure and heat. 

## 4. Conclusions

A novel flexible nanocomposite’s synthesis, manufacturing, mechanical, and electrical testing were reported in this paper. The developed nanocomposites had an extremely high maximum elongation up to 244% tensile strain and were proven durable over cyclic tests. Moreover, the dispersion of MWCNTs at only 0.25 wt.% resulted in a reliable piezoresistive-based strain sensing capability. The gauge factors of the tested samples were about 1.0–2.0, depending on the applied strain ranges. Additionally, the DLP-based 3D printing process was modified to allow quick printing comparable to virgin photopolymers without adding any extra photo-initiator. The viscosity of the nanocomposite responded well to the increased printing temperature to enable easy flow and detail retention during the printing process. High magnification optical imagery demonstrated the cause of anisotropic characteristics of the printed samples where MWCNTs aligned in the horizontal plane. The alignment of MWCNT nanoparticles allowed for the improved strain sensing response when pulling along the vertical axis. Last but not least, wearable sensors were customized, and 3D printed to demonstrate the sensor’s ability to be used as a skin-attachable and wearable sensor for the detection and monitoring of human kinetic motions. A MATLAB code was developed to automatically smooth and detect key sensor signals and features in the data to highlight different points of motion. Overall, the developed novel nanocomposite demonstrated the ability to use low-cost resins and 3D printing systems to develop customized and wearable strain sensing systems applicable to broad engineering applications including in situ health monitoring, patient rehabilitation, prosthetics and robotics, human–machine interfaces, and virtual and augmented reality devices. 

## Data Availability

The data that support the findings of this study are available on request from the corresponding author, Yingtao Liu.
